# Personalized Post-Stroke Rehabilitation in a Rural Community: A Pilot Quasi-Experimental Study on Activities of Daily Living and Disability Outcomes Using Participatory Action Research

**DOI:** 10.3390/healthcare13111275

**Published:** 2025-05-28

**Authors:** Mallika Piromboon, Kwanjai Suebsunthorn, Kanokwan Wisaddee, Le Ke Nghiep, Kukiat Tudpor

**Affiliations:** 1 Department of Rehabilitation Medicine, Nachuak Hospital, Maha Sarakham 44170, Thailand; chompoo.pn@gmail.com; 2 Department of General Medicine, Nachuak Hospital, Maha Sarakham 44170, Thailand; khuanwjai40150@hotmail.com (K.S.); kanokwan_oom@hotmail.com (K.W.); 3 Vinh Long Department of Health, Vinh Long 85000, Vietnam; lekenghiep@gmail.com; 4 Public Health and Environmental Policy in Southeast Asia Research Cluster (PHEP-SEA), Mahasarakham University, Maha Sarakham 44150, Thailand; 5 Faculty of Public Health, Mahasarakham University, Maha Sarakham 44150, Thailand

**Keywords:** neurorehabilitation, intermediate care, Barthel index, activities of daily living, stroke

## Abstract

**Background**: Early rehabilitation is crucial for predicting post-stroke outcomes. In rural Thailand, previous works identified limited access to prompt rehabilitation services, discontinuity of home visits, and a lack of interdisciplinary management, hindering comprehensive resolution. **Objective**: This participatory action research-based pilot quasi-experimental study investigated the effects of personalized intermediate care (IMC) programs led by physical therapists on clinical outcomes in post-ischemic stroke older adults living in rural areas. **Methods**: Participatory stakeholders (two physical therapists, a physician, a nurse, and a nutritionist) convened to coordinate with relevant stakeholders (community leaders, village health volunteers (VHVs), and family caregivers (CGs)). Thirty-four acute post-stroke patients were included in the study. The interventions consisted of three action research cycles (planning, action, observation, and reflection) of home-based neurorehabilitation and comprehensive treatments by a healthcare professional network for six months and another six-month follow-up. The primary outcome was the Barthel index for activities of daily living (BI-ADL). The modified Rankin scale (mRS) was a secondary outcome for assessing disability levels. **Results**: Results showed that the BI-ADL gradually and significantly increased from a baseline median (IQR) of 55 (15) to 100 (20) after 6 months (*p* < 0.05). This improvement of the BI-ADL was maintained after 12 months (100 (15)). Furthermore, the mRS at 6 months post-discharge reduced considerably from the first month of rehabilitation (*p* < 0.05). **Conclusions**: In conclusion, the early and continuous personalized IMC rehabilitation program effectively enhanced ADL and reduced disability levels and should be disseminated to the community.

## 1. Introduction

Stroke is a primary cause of disability worldwide [1]. Following the surgical and pharmacological treatments, many stroke survivors are referred to an inpatient rehabilitation department for continued and intensive therapy in the early stages of recovery. Inpatient rehabilitation aims to maximize neurological and functional recovery so patients can reintegrate into the community after discharge [2]. However, not everyone has the same ability to recover. After initial therapy, patients experience widely different degrees of function, with some returning to premorbid function and others sustaining significant deficits that necessitate extra care at home or in the community [3].

In Thailand, stroke is the leading cause of adult impairment and the leading cause of mortality, especially for older patients. According to the 2014 Thailand Burden of Disease study, stroke was the primary cause of death in seven out of thirteen Ministry of Public Health (MOPH) areas, or two out of four regions. During that time, around 250,000 strokes occurred annually, while over 50,000 people died from them. Between 2008 and 2012, the mortality rate increased from 20.8 to 30.7 strokes per 100,000 people. Therefore, in 2017, an intermediate care (IMC) program for patients during transitional phases was founded in Thailand [4]. Subacute conditions refer to situations where patients have transitioned beyond the critical stage and exhibit persistent abnormalities in certain bodily functions, resulting in limitations in their activities of daily living [5,6]. Therefore, continuous care to improve physical functionality and prevent complications becomes imperative, as it can significantly enhance patients’ ability to lead independent daily lives while reducing the risk of complications.

Continuous IMC can also be a preventive measure against complications that may lead to long-term disabilities [7]. Over the past three years (from October 2019 to January 2023), Nachuak Hospital has been developing a program for providing IMC services. During this period, a total of 123 IMC patients were registered. Through post-discharge patient follow-ups, variations in the abilities of patients to resume their daily lives were observed. Some patients passed away after discharge, while others did not receive continuous medication rehabilitation or remained bedridden and developed recurring disabilities over time. Studies conducted in Thailand have reported that the mortality rate among patients with acute stroke within 30 days after hospital discharge is 16.63% [8].

Furthermore, a significant proportion of stroke patients continue to experience residual disabilities. In the U.S., over 80% have lower limb dysfunction, with 25% still disabled after rehabilitation. In China, 85% initially present with upper limb dysfunction, and 30–36% show ongoing impairments six months later [9]. In Thailand, 30% could not walk independently, 19% had speech problems, and 35% had psychological symptoms within 6 months [10]. The findings from these practical experiences and academic reports have driven the development of an IMC patient care program. This program aims to align with the policies of the Ministry of Public Health, ensuring effective and continuous care for IMC patients. The ultimate goal is to enable IMC patients to lead lives as close to normalcy as possible while reducing the prevalence of bedridden patients and ongoing disabilities.

Having meticulously analyzed and implemented physical therapy services, we have encountered significant impediments. These primarily involve patients lacking consistent caregiver support, exhibiting fragmented adherence to treatment and follow-up protocols, and contributing to interrupted data collection processes. Critically, these multifaceted challenges extend beyond the direct remit of physical therapy intervention. Consequently, the research team convened to formulate comprehensive guidelines for the collaborative management of integrated care patients. This initiative is specifically designed to proactively address these issues, aiming to deliver more holistic and seamlessly integrated care, thereby paving the way for developing a robust interdisciplinary care program.

The research question was how implementing collaborative, comprehensive, integrated care guidelines affects caregiver support, treatment adherence, follow-up, and data completeness versus prior physical therapy delivery.

This participatory action research aims to determine the effects of a developed personalized intermediate care rehabilitation program for post-stroke patients in a rural community. This study hypothesized that the program would improve patients’ activities of daily living and reduce their disability, with the optimal goal of achieving full independence in daily activities.

## 2. Materials and Methods

### 2.1. Research Framework, Design, and Settings

The Ministry of Public Health established a policy to develop the IMC service system. This policy aimed to provide care for patients who had recovered from a critical condition, promote their return to everyday life, reduce central hospital overcrowding, and enhance the overall quality of healthcare services. Our present study involves Phase 3, which continues previous preliminary work in Phases 1 and 2.

#### 2.1.1. Phase 1: Analysis of Services

As part of this initiative, Nachuak Hospital started a healthcare unit for IMC patients referred from other general provincial hospitals in 2021. However, having analyzed the clinical outcomes, we found that after these patients returned home, they often experienced complications, such as pressure sores, urinary tract infections, joint contractures, and even mortality. In response to these challenges, the research team conducted a three-month service assessment and analyzed the situation with healthcare professionals. This analysis led to the development of Phase 2.

#### 2.1.2. Phase 2: Physical Therapy Services in the Community

Phase 2 commenced in January 2022. Physical therapists proposed an action plan for services outside the hospital unit under the “Physical Therapy for Community” project. All IMC patients returning to the hospital received physical therapy services. Subsequently, continuous home visit and rehabilitation plans were implemented, involving monthly assessments of patients’ conditions and providing rehabilitation services following professional physical therapy standards. This program continued until the end of the fiscal year 2022, September 2022. It was observed that patients’ mobility improved, as assessed by the BI-ADL score. However, there were still limitations, as patients and healthcare providers faced several challenges, including (1) patients without caregivers, (2) patients not receiving continuous medications, (3) patients with oral health issues, (4) patients not registered as intermediate care receivers, and not receiving followed-ups, (5) patients’ relocation, and (6) sporadic data collection. These issues were beyond the scope of physical therapy. Therefore, the research team convened and jointly developed guidelines for IMC patient management to address these issues, aiming to provide more comprehensive and integrated care, leading to the development of Phase 3.

#### 2.1.3. Phase 3: Development of the Interdisciplinary Intermediate Care Rehabilitation Program

**Figure 1:**
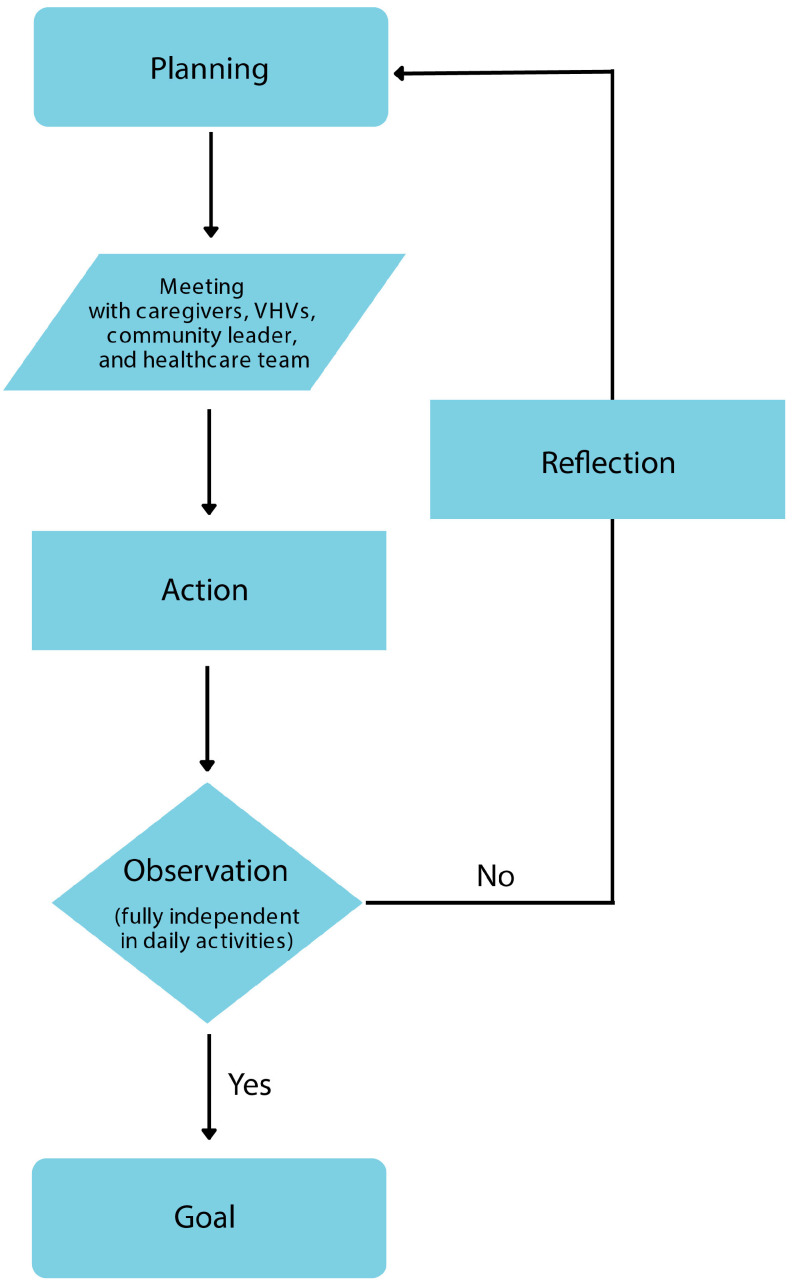
Action research cycle for intermediate care rehabilitation program development. Each cycle has four steps (planning, action, observation, and reflection). A research team of two physical therapists, a physician, a nurse, and a nutritionist met during the planning phase to coordinate data collection with pertinent parties (community leaders, VHVs, and family CGs) and discussed protocols for IMC patient rehabilitation and created a care plan. Home visits were carried out following the predetermined strategy for the action phase. Using information from the planning step, physical therapists evaluated patients’ conditions and administered physical therapy interventions during the action phase. The BI-ADL and mRS were assessed throughout the observation period. The research team addressed obstacles and modified the operational plan (re-planning) as part of a reflective process in the cycle’s last phase. Over six months, these cycles were repeated three times.

This one-group pretest–posttest quasi-experimental design within an action research framework was conducted in rural areas of Maha Sarakham Province, Thailand, from October 2022 to January 2024. The study was approved by the Nachuak Hospital Human Research Ethical Committee, Maha Sarakham Province (EC number 2565-022) and followed the principles of the Declaration of Helsinki. All patients or their caregivers provided informed consent. This study employed four main steps of a research cycle (planning, action, observation, and reflection, PAOR). In the planning step, a research team (two physical therapists, a physician, a nurse, and a nutritionist) convened to establish coordination with relevant stakeholders (community leaders, VHVs, and family CGs) for data collection. They reviewed guidelines for rehabilitating IMC patients and developed a recovery plan. For the action step, home visits were conducted per the established plan. Physical therapists assessed patient conditions and provided physical therapy interventions based on inputs from the planning step and continual consultation with the team members. During the observation period, assessments using the Barthel index for activities of daily living (BI-ADL) and modified Rankin scale (mRS) [11] were conducted before patients were discharged from the community hospital. In the final step of the cycle, the research team engaged in a reflective process, addressing challenges encountered and adjusting the operational plan (re-planning). These cycles were repeated 3 times in six months (Figure 1). The BI-ADL and mRS assessments were performed twice each cycle (a total of six repeats—months 1–6) and at a 12-month follow-up.

### 2.2. Infrastructural Support and Clinical Services

An intramural service unit was constituted with a committee of healthcare professionals to ensure that IMC patients receive standardized and specialized care. Patients were monitored from the moment they met the criteria for IMC, such as those with conditions like stroke, spinal cord injury (SCI), traumatic brain injury (TBI), and fragility fractures in patients aged 50 and above. Following confirmation of the diagnosis from general hospitals, data were shared with researchers to enhance follow-up and accessibility to services. The hospital provided a 2-bed unit within the inpatient building to accommodate patients who return for treatment. IMC patients received healthcare services from various healthcare professionals at an IMC clinic. The clinic dedicated to IMC patients operates on Wednesdays.

Patients received physical therapy and traditional Thai medicine services during weekday office hours. The patients were screened for chronic diseases and sought treatment at the hospital for stroke risk factors. For an extramural service unit, the outpatient department established a “Rehabilitation for Community” project. This plan includes home visits for registered IMC patients and follow-ups for the patients referred to a larger tertiary hospital with BI-ADL scores below 75. The physical therapy program included personalized sensorimotor, proprioception, task-specific repetitive, and balance training therapy. The research team coordinates with relevant healthcare professionals, including physical therapists, nutritionists, traditional Thai medicine practitioners, professional nurses from public health centers, and volunteers in the community. In cases where complications are identified, the attending physician is informed, and a further care plan is developed. The patients were referred for consultation with a dentist regarding oral health issues. Additionally, we built a community-based care network involving VHVs and community leaders to facilitate patient transport and support regarding healthcare and community reintegration. These measures aimed to create a comprehensive and collaborative care system for IMC patients within and outside the hospital.

### 2.3. Inclusion and Exclusion Criteria

The study included patients with stroke who were admitted to Nachuak Hospital at age ≥ 18 years with medical and neurological sign stability, could sit for at least 30 min, had functional impairment (BI-ADL score < 75), could follow a 1-step command or had caregivers who could understand and participate in the rehabilitation program, and had willingness to participate. The patients with neurological conditions (transient ischemic attack (TIA) or cervical myelopathy), psychiatric disorders, and pre-existing conditions, including head injuries, tumors, and central nervous system infections, were excluded. Additionally, corresponding stakeholders (community leaders, VHVs, and family CGs) were invited to the study.

### 2.4. Research Instruments

We used three research instruments: IMC patient care guidelines, which outline the care plan from the beginning of service provision to patient discharge from continuous recovery obtained from collaborative meetings with healthcare professionals; a home-based rehabilitation action plan, an integral part of the research process; and assessment forms for data collection (BI-ADL and mRS).

#### 2.4.1. Barthel Index for Activities of Daily Living Score Assessment Form

The BI-ADL score assessment form (Thai version) was used to assess patients’ abilities to perform ADL. This assessment tool provided a reliable measurement with a reliability level of 0.88 when evaluating post-stroke patients [12]. The scoring criteria were 0–20 (unable to perform daily activities), 25–45 (minimal ability to perform daily activities), 50–70 (moderate ability to perform daily activities), 75–95 (high ability to perform daily activities, and 100 (fully independent in daily activities).

#### 2.4.2. Modified Rankin Scale Assessment Form

The mRS was used to evaluate the level of disability based on the degree of assistance required for various tasks and activities. It is easy to use and allows for quick assessment. The reliability of mRS ranges from 0.81 to 0.95 [13]. The scoring criteria for the mRS are as follows: 0, no symptoms; 1, no significant disability and inability to perform activities normally; 2, slight disability and inability to perform previous activities without assistance; 3, a moderate disability that requires some assistance but can walk without aid; 4, moderately severe disability and can walk with assistance and cannot self-care; 5, severe disability, bedridden, requiring continuous care; and 6, deceased.

### 2.5. Statistical Analyses

Statistical analyses were conducted employing IBM SPSS Statistics, version 25. The Kolmogorov–Smirnov test was utilized to ascertain data normality. Subsequently, the non-parametric Friedman ANOVA was implemented to evaluate variations across different time points. Where significant differences were identified, post hoc pairwise comparisons were performed using Wilcoxon signed-rank tests, incorporating a Bonferroni correction to mitigate the risk of Type I errors. A *p*-value below 0.05 was predetermined as the threshold for statistical significance.

## 3. Results

### 3.1. Demographic Characteristics

The primary patient data for this study consisted of 31 IMC ischemic stroke patients diagnosed at a larger tertiary hospital in the same province (Mahasarakham Hospital) and registered as IMC patients at Nachuak Hospital. The mean age was 62.7 ± 13.1 years, with 21 females (67.5%) and 10 males (32.5%) with an approximately 7-day post-stroke period. More than 50% had a monthly income above the cut-off point for poverty (<5000 THB) [14]. Other details are presented in Table 1. A baseline of neurological assessments is depicted in Table 2. The assessments include sensory assessments (light touch, pinprick, and proprioception), motor coordination, sitting and standing balance (static and dynamic), ambulation ability, and neglect syndrome.

### 3.2. The Developed Interdisciplinary Intermediate Care Rehabilitation Program

A committee for IMC patients was established at the hospital to ensure comprehensive and integrated care. After three development cycles, the committee agreed that the program consisted of four stages: preventive screening, early diagnosis and treatments, early rehabilitation, and networking. In the first stage, individuals with chronic non-communicable diseases were screened for co-morbidities and stroke risk. The patients and caregivers were educated about the causes of stroke and the early recognition of the signs of stroke. In the second stage, when patients met the criteria above, the emergency and nursing units referred them to general hospitals for detailed medical examinations, diagnoses, and pharmacological treatments by physicians. When the patients were categorized as IMC cases, they were further treated in the inpatient wards for 3–7 days. At this point, nutritionists were consulted to assess nutritional intake and provide dietary recommendations. Later, physical therapists were consulted to plan an early rehabilitation program with interdisciplinary professionals. In the last stage, continuous extramural networking with community health promotion officers and VHVs was conducted with home visits using a Continuing of Care Region 9 (COCR9) system involving hospital staff.

### 3.3. The Intermediate Care Rehabilitation Program Improved the Activities of Daily Living Score

Descriptive statistics in Table 3 show changes in BI-ADL scores over 1 to 6 months of the intervention and the follow-up at 12 months. A Friedman test was conducted to examine whether there were differences in BI-ADL scores across seven time points: 1 month, following 2-, 3-, 4-, 5-, and 6-month intervention periods, and at a 12-month follow-up. The results indicated a statistically significant difference in BI-ADL scores, χ^2^(6) = 147.327, *p*-value < 0.001. Therefore, we rejected the null hypothesis and concluded that BI-ADL scores differed significantly across the seven measurement times. The mean ranks for BI-ADL scores at each time point were examined to explore these differences further. The mean ranks were as follows: baseline (month 1) = 1.13, month 2 = 2.18, month 3 = 3.58, month 4 = 4.68, month 5 = 5.18, month 6 = 5.66, and month 12 = 5.60 (Table 3).

### 3.4. Intermediate Care Rehabilitation Program Reduced Disability Level

Table 4 presents descriptive statistics illustrating how modified Rankin scale (mRS) scores changed from the beginning of the intervention (1 month) through the 6-month intervention period and at the 12-month follow-up. A Friedman test revealed a statistically significant difference in mRS scores across the seven measurement points (1, 2, 3, 4, 5, and 6 months of intervention, and a 12-month follow-up), as indicated by χ^2^(6) = 144.994 and a *p*-value < 0.001. Consequently, we concluded that there were significant changes in mRS scores over these time points. Further analysis of the mean ranks for mRS at each time point showed a trend of decreasing disability, with the highest mean rank at baseline (6.81) and progressively lower mean ranks during the intervention (ranging from 5.73 at month 2 to 2.47 at month 6), with a slight increase at the 12-month follow-up (2.52), as detailed in Table 4.

## 4. Discussion

Discharge after stroke is a major challenge, involving lasting impairments, new medications, rehabilitation, and dietary changes. Over 50% of patients leave the hospital with limited understanding of deficits, hindering secondary prevention and recovery [15]. Post-acute care gaps impede risk factor management and recovery preparation, leading to poor outcomes [16]. Within 90 days, one-fourth are readmitted and lack medication adherence, over 50% have uncontrolled blood pressure, and patients are sedentary 78% of the time, with a 73% fall rate [17]. Consequently, stroke recurrence and disability prevalence remain high, escalating post-acute recovery [18]. This pilot quasi-experimental study demonstrated that the IMC program under the PAOR approach is efficacious in improving ADL and reducing disability of post-stroke patients. The following findings support our statement: significantly increased BI-ADL and reduced mRS scores after 1 month, with steady effects until the end of the follow-up period.

The development of the IMC patient care system received comprehensive support from hospital administrators, interdisciplinary professionals, and the community. During weekday hours, patients received physical therapy and traditional Thai medicine and were screened and treated for stroke risk factors at the hospital. A “Rehabilitation for Community” project was established to extend care beyond the hospital, offering home visits for registered patients and follow-ups for those with lower functional scores referred to a tertiary center. A previous study in China demonstrated that the transitional and home healthcare models designed to cater to individuals necessitating consistent nursing support following hospital discharge improved the BI score after 1 month [19]. Typically, such care is delivered by family members, though professional in-home caregivers may occasionally be involved. This integrated approach addresses the ongoing care needs of patients requiring regular assistance by health professionals beyond the acute hospital setting. More recently, the hospital community-integrated service model (HCISM) has been designed to address these needs by improving post-discharge quality of life, alleviating family burdens, and enhancing community medical staff capabilities. This study investigated the impact of the HCISM on home rehabilitation for disabled elderly stroke patients by comparing a routine intervention group with a group receiving the HCISM intervention. The findings revealed that after three months, the HCISM group demonstrated significantly improved modified BI scores, medication adherence, self-efficacy, and reduced anxiety and depression compared to the control group [20]. Similar to our study, a study in Thailand showed that early physical therapist-led home-based care was associated with statistically significant improvements in BI and mRS scores for participants in the home-based rehabilitation group [21].

We can highlight four key points for successful rehabilitation: a comprehensive IMC program development, preventive stroke risk screening and recognition, early diagnosis and referral, interdisciplinary IMC implementation, and continuous extramural networking.

First, even though the IMC program was introduced in 2017, our hospital committee was the first to develop a comprehensive care program for IMC patients with a structured stage: preventive screening, early diagnosis and treatments, early rehabilitation, and networking. According to evidence-based clinical practice recommendations, stroke rehabilitation is an ongoing process that begins when the patient first exhibits impairments and may require ongoing care for the remainder of their life [22]. To achieve this, rehabilitation encounters must be established appropriately to identify comprehensive therapies at every level of care with an early approach for inpatient and outpatient care, subsequent home-based rehabilitation with community integration, and prolonged and consistent rehabilitation [23].

Secondly, preventive screening focuses on patients with co-morbidities, especially hypertension, diabetes, atrial fibrillation, and hyperlipidemia [24,25,26]. In practice, we pay special attention to patients who are not compliant with the medications and lifestyle modification advice [26]. The individuals with chronic diseases at risk for stroke and their caregivers were educated on stroke prevention and recognition. Regarding early diagnosis and treatments, in 2007, the Thai medical system initiated the stroke fast track system—intravenous recombinant tissue plasminogen activator (rt-PA) treatment within 4.5 h of the onset of the ischemic stroke, the most common type of stroke in Thailand (80%) [27].

Thirdly, in this present study, we emphasized an early consultation for rehabilitation. Patients meeting specific criteria were referred to physical therapists. The IMC patients received inpatient treatment for 3–7 days, where interdisciplinary professionals collaborated on rehabilitation planning. The rehabilitation program included personalized sensorimotor, proprioception, task-specific repetitive, and balance training therapy [28,29]. The BI-ADL is widely used to assess impairment and monitor stroke patients’ functional recovery since it is regarded as a valid disability scale [30]. This present study found that BI-ADL scores significantly increased after the IMC program over 6 months and stabilized over another 6-month follow-up period. A previous study showed that the participants receiving a neurodevelopmental treatment for 1 h/day, 6 days a week, for 6 months did not improve upper extremity motor functions [31]. This unobserved leg motor improvement might be due to the lack of task-specific repetitive training, a pivotal component for motor relearning in post-stroke patients [32]. Hence, we suggest that task-specific repetitive training is indispensable for post-stroke rehabilitation programs. We also found that after the IMC program, the mRS score was significantly reduced from baseline over 6 months and the follow-up. The mRS is typically used to evaluate disability following an acute stroke at a particular moment, such as 3 or 12 months post-stroke [33]. It has been shown that repeated-measure mRS data collection helps assess functional recovery over time better than a single measurement [34]. According to the mRS ratings, the levels of disability in our patients reduced from moderate (approximately scale 4) to no significant disability (approximately scale 1), indicating the effectiveness of the program

Lastly, networking should be available in the hospital and with specialists, such as primary care providers, rehabilitation specialists, care transitions, and telemedicine in other health settings [35]. Lastly, continuous home visits utilizing the COCR9 system ensure ongoing care and support for patients after discharge. Interestingly, a previous study in Thailand shows that home-based physical therapy in chronic stroke patients resulted in significant improvements in voluntary movement and postural balance but not ADL [36]. This finding signifies the importance of the early rehabilitation program. Altogether, this program allows for a seamless connection between public health and local healthcare, as there are clear and accessible service programs. Lessons learned from this study include the value of (1) developing a service system that relies on teamwork and a well-planned systematic approach, (2) effective coordination and integration with the community, and (3) rapid patient access that helps reduce bedridden conditions and disabilities. The success factors consist of support from hospital administrators, teamwork within the hospital and the network of healthcare professionals, including the sub-district health promotion hospital and village health volunteers, the knowledge and abilities of interdisciplinary professionals who plan together, and collaboration between patients and caregivers. Our findings align with the previous statement that the post-stroke rehabilitation outcomes varied with age, ambulation status before stroke, stroke severity score at admission, sitting balance, and motivation for rehabilitation programs, emphasizing the importance of networking with caregivers and VHVs [37].

This study generally observed a trend of more favorable functional outcomes in patients presenting with what clinically appear to be smaller subcortical strokes, even without definitive classification via CT imaging. This finding aligns with existing literature suggesting that the location and extent of the initial ischemic insult significantly influence recovery trajectories. Lacunar strokes, caused by occlusions of small penetrating arteries, are often associated with specific, more predictable neurological deficits and potentially better long-term outcomes [38]. Non-lacunar strokes, involving larger arteries, can lead to more widespread and severe deficits, potentially impacting functional recovery more significantly [39]. While we lacked CT confirmation to categorize strokes into lacunar and other subtypes, our personalized IMC programs were designed to be responsive to the specific neurological deficits and observed recovery patterns of each individual. This patient-centered approach allowed us to tailor interventions based on the actual functional impairments and progress, acknowledging the inherent variability in recovery following acute ischemic stroke, regardless of precise subtyping based on imaging. Further research, incorporating neuroimaging for detailed lesion characterization, would enhance our understanding of the differential impacts of specific stroke subtypes on the effectiveness of personalized rehabilitation strategies within our community.

Our study acknowledges certain limitations, notably the incomplete evaluation of all facets within the stroke rehabilitation quality improvement project [40]. Specifically, we did not comprehensively assess the intervention through the lens of the Donabedian triad, a well-established framework for evaluating healthcare quality articulated by Avedis Donabedian [41]. This triad encompasses three critical dimensions: structure, which pertains to the resources underpinning care delivery, including infrastructure, equipment, staffing, and financial provisions; process, which encompasses the actual delivery of care, encompassing patient–provider interactions, adherence to protocols, and the treatments administered; and outcomes, which reflect the impact of care on patients’ health status, such as mortality rates, morbidity, and patient satisfaction levels. In the previous work, Alcock and colleagues showed that the Donabedian quality framework-based quality improvement initiative reduced door-to-needle times for thrombolysis, which were associated with decreased in-hospital mortality and a greater proportion of patients being discharged to favorable locations, underscoring the importance of timely stroke care [42]. More recently, Blaquera and colleagues demonstrated that nurse-coordinated home-based rehabilitation using the Delphi technique and Donabedian model revealed key elements such as interdisciplinary teams, culturally responsive care processes, and patient-centered outcomes focused on daily activities and quality of life, providing crucial guidance for improving community-based stroke care in the Philippines [43]. Future research endeavors could fruitfully employ the Donabedian triad to broaden the perspectives within post-stroke rehabilitation research and provide a more holistic evaluation of quality improvement initiatives in Thailand’s contexts.

Quasi-experimental designs offer notable advantages, particularly when implementing randomized controlled trials, such as those investigating beneficial interventions, which can prove either impractical or ethically problematic. Specifically, the random assignment of post-stroke patients to a non-treatment control group could ethically compromise or unduly postpone their potential for recovery. A prospective study by Dromerick about a sensitive or optimal period for intensive motor rehabilitation, 60 to 90 days after stroke, with findings of no significant effect with a later intervention (≥6 months), highlights the importance of timely intervention within a specific window [44]. Nevertheless, these designs are not without limitations. The absence of random assignment introduces the potential for confounding variables, and evaluating the intervention’s impact relies heavily on comparisons with a pre-existing baseline. Further studies may apply alternative study designs in post-stroke rehabilitation, such as comparing active interventions. Instead of a no-treatment control, the study could compare two or more promising rehabilitation approaches. Additionally, the intervention can be compared to the standard rehabilitation practices already in place (the usual care control groups).

## 5. Conclusions

In summary, early rehabilitation for post-stroke outcomes in rural Thailand, where access to rehabilitation services is limited, was studied. The research explored the impact of a personalized IMC program led by physical therapists for older adults recovering from strokes. The program included home-based neurorehabilitation and telerehabilitation over six months, followed by another six-month follow-up. Key findings include a significant improvement in the BI-ADL after 1 month and throughout six months, and reduced disability levels measured by the mRS among the post-stroke patients. The study concluded that early, continuous IMC rehabilitation improves daily living functions and reduces disability, recommending its implementation in rural communities.

## Figures and Tables

**Table 1:** Basic characteristics of post-stroke patients.

Characteristics	*N* (%) or Mean ± SD
Male	14 (45.2)
Female	17 (54.8)
**Age**	
<60 years	20 (64.5)
≥60 years	11 (35.5)
**Education**	
Illiterate/primary school	17 (54.8)
Secondary school/higher	14 (45.2)
**Marital status**	
Married	15 (48.4)
Single, divorced, widowed	16 (51.6)
**Occupation**	
Farmer	20 (64.5)
Other works	11 (35.5)
**Income per month** (THB)	
<5000 (<150 USD)	15 (48.4)
≥5000 (≥150 USD)	16 (51.6)
**Post-stroke duration** (days)	7 ± 0.5

**Table 2:** Baseline of neurological signs of post-stroke patients.

No.	Sensory Assessment			Motor Coordination	Balance				Ambulation Ability	Neglect Syndrome
	Light touch	Pinprick	Proprioception		Sitting		Standing			
					Static	Dynamic	Static	Dynamic		
1	Normal	Normal	Fair	Poor	Good	Fair	Poor	Poor	Fair	Negative
2	Normal	Normal	Lost	Lost	Poor	Poor	Poor	Poor	Lost	Negative
3	Normal	Normal	Fair	Poor	Good	Good	Poor	Poor	Fair	Negative
4	Normal	Normal	Poor	Fair	Poor	Poor	Poor	Poor	Poor	Negative
5	Normal	Normal	Poor	Lost	Poor	Poor	Poor	Poor	Lost	Negative
6	Normal	Normal	Poor	Lost	Poor	Poor	Poor	Poor	Lost	Negative
7	Impaired	Impaired	Lost	Lost	Lost	Lost	Lost	Lost	Lost	Positive
8	Normal	Normal	Poor	Lost	Lost	Lost	Lost	Lost	Lost	Negative
9	Normal	Normal	Poor	Lost	Fair	Lost	Lost	Lost	Lost	Negative
10	Normal	Normal	Poor	Lost	Poor	Lost	Lost	Lost	Lost	Negative
11	Normal	Normal	Poor	Lost	Poor	Lost	Lost	Lost	Lost	Negative
12	Normal	Normal	Poor	Lost	Fair	Lost	Lost	Lost	Lost	Negative
13	Normal	Normal	Poor	Lost	Poor	Lost	Lost	Lost	Lost	Negative
14	Normal	Normal	Good	Good	Good	Fair	Fair	Poor	Fair	Negative
15	Impaired	Impaired	Lost	Lost	Lost	Lost	Lost	Lost	Lost	Negative
16	Normal	Normal	Fair	Lost	Good	Fair	Fair	Poor	Poor	Negative
17	Normal	Normal	Poor	Lost	Fair	Fair	Lost	Lost	Poor	Negative
18	Normal	Normal	Poor	Lost	Poor	Poor	Lost	Lost	Lost	Negative
19	Normal	Normal	Poor	Lost	Poor	Poor	Lost	Lost	Lost	Negative
20	Normal	Normal	Fair	Fair	Fair	Fair	Fair	Poor	Poor	Negative
21	Normal	Normal	Poor	Lost	Fair	Fair	Poor	Poor	Poor	Negative
22	Normal	Normal	Good	Good	Good	Fair	Fair	Fair	Fair	Negative
23	Impaired	Normal	Lost	Lost	Lost	Lost	Lost	Lost	Lost	Negative
24	Normal	Normal	Poor	Lost	Poor	Poor	Lost	Lost	Lost	Negative
25	Normal	Normal	Poor	Lost	Fair	Fair	Fair	Poor	Poor	Negative
26	Normal	Normal	Poor	Lost	Lost	Lost	Lost	Lost	Lost	Negative
27	Impaired	Impaired	Lost	Lost	Lost	Lost	Lost	Lost	Lost	Negative
28	Normal	Normal	Poor	Lost	Poor	Poor	Lost	Lost	Lost	Negative
29	Normal	Normal	Good	Fair	Good	Good	Good	Fair	Fair	Negative
30	Normal	Normal	Poor	Lost	Poor	Poor	Lost	Lost	Lost	Negative
31	Normal	Normal	Lost	Lost	Lost	Lost	Lost	Lost	Lost	Negative

**Table 3:** Descriptive and Friedman’s ANOVA statistics of the Barthel index for activities of daily living score.

Barthel Index for Activities of Daily Living Score					
Time Point	*N*	Mean (SD)	Median (IQR)	Range (Min–Max)	Mean Rank *
1-month	31	51.29 (16.33)	55 (15)	0–75	1.13 ^a^
2-month	31	67.74 (14.82)	75 (20)	20–90	2.18 ^b^
3-month	31	79.84 (18.10)	85 (15)	25–100	3.58 ^c^
4-month	31	85.65 (18.47)	90 (35)	35–100	4.68 ^d^
5-month	31	88.39 (17.05)	100 (25)	45–100	5.18 ^e^
6-month	31	90.97 (15.24)	100 (20)	45–100	5.66 ^f^
12-month	31	90.81 (18.67)	100 (15)	25–100	5.60 ^d,e,f^

* Wilcoxon signed-rank test post hoc pairwise comparisons with a Bonferroni correction. Different letters (a–f) indicate significant differences between time points (*p* < 0.05).

**Table 4:** Descriptive and Friedman’s ANOVA statistics of the modified Rankin scale score.

Modified Rankin Scale Score					
Time Point	*N*	Mean (SD)	Median (IQR)	Range (Min–Max)	Mean Rank *
1-month	31	4.19 (0.749)	4 (1)	3–5	6.81 ^a^
2-month	31	3.16 (1.098)	3 (2)	1–5	5.73 ^b^
3-month	31	2.26 (1.182)	2 (2)	1–5	4.37 ^c^
4-month	31	1.77 (1.230)	1 (2)	0–5	3.32 ^d^
5-month	31	1.55 (1.287)	1 (2)	0–5	2.79 ^d^
6-month	31	1.42 (1.285)	1 (2)	0–4	2.47 ^d^
12-month	31	1.35 (1.279)	1 (1)	0–5	2.52 ^d^

* Wilcoxon signed-rank test post hoc pairwise comparisons with a Bonferroni correction. Different letters (a–d) indicate significant differences between time points (*p* < 0.05).

## Data Availability

Data are available upon request.
